# Anxiety makes time pass quicker: neural correlates

**DOI:** 10.1093/scan/nsag006

**Published:** 2026-02-06

**Authors:** Ioannis Sarigiannidis, Karel Kieslich, Christian Grillon, Monique Ernst, Jon P Roiser, Oliver J Robinson

**Affiliations:** UCL Institute of Cognitive Neuroscience, University College London, London, WC1N 3AZ, United Kingdom; Institute of Psychiatry, Psychology & Neuroscience (IoPPN), King’s College London, 16 De Crespigny Park, London SE5 8AB, United Kingdom; UCL Institute of Cognitive Neuroscience, University College London, London, WC1N 3AZ, United Kingdom; National Institutes of Mental Health, Bethesda, MD 20892, United States; National Institutes of Mental Health, Bethesda, MD 20892, United States; UCL Institute of Cognitive Neuroscience, University College London, London, WC1N 3AZ, United Kingdom; UCL Institute of Cognitive Neuroscience, University College London, London, WC1N 3AZ, United Kingdom; UCL Department of Clinical Education and Health Psychology, University College London, London, WC1E 6BT, United Kingdom

**Keywords:** anxiety, threat-of-shock, cingulate cortex, time perception, fMRI

## Abstract

Anxiety can be adaptive, but at a cost. One theory suggests that whilst anxiety promotes harm-avoidant cognitive processing, it impairs concurrent (non-harm-related) processing by commandeering finite neurocognitive resources. Our previous work has shown that anxiety reliably ‘speeds up time’, promoting temporal underestimation, possibly due to a loss of temporal information. Whether this results from anxiety overloading neurocognitive systems involved in time processing remains unclear. Here, we examined whether anxiety and time processing overlap, particularly in regions of the cingulate cortex. Across two studies (an exploratory Study 1, *N* = 13, informing a pre-registered Study 2, *N* = 29), we combined a well-established anxiety manipulation (threat of shock) with a temporal bisection task while participants underwent fMRI. Consistent with our previous findings, time was perceived to pass more quickly under anxiety. Anxiety induction led to widespread activation in the cingulate cortex, while perceiving longer intervals was associated with more circumscribed activation in a mid-cingulate region. Importantly, conjunction analysis revealed convergence between anxiety and time processing in the insula and mid-cingulate cortex. These results tentatively support the idea that anxiety overloads already-engaged neural resources. In particular, overloading mid-cingulate capacity may drive emotion-related changes in temporal perception, consistent with its hypothesized role in mediating responses to anxiety.

## Introduction

Anxiety profoundly alters how we perceive the world (Grupe and Nitschke 2013, Robinson et al. 2013), promoting harm-avoidant behaviours. Anxious individuals tend to pay more attention to threatening stimuli (e.g. attentional bias: Cisler and Koster 2010, Robinson et al. 2014, Van Bockstaele et al. 2014), interpret ambiguous information as threatening (e.g. interpretation bias: Wilson et al. 2006) and overestimate the probability and personal cost of negative events (e.g. judgement bias: Mitte 2007, Charpentier et al. 2017, Aylward et al. 2019). While previous behavioural and neuroimaging work has mainly focused on how anxiety influences emotional information (Mathews et al. 1997, Bar-Haim et al. 2007, Cisler and Koster 2010, Carlisi and Robinson 2018), less research has been conducted on how anxiety influences non-emotionally valanced information (Robinson et al. 2013).

In a series of prior studies we demonstrated that anxiety (induced in healthy individuals using threat of unpredictable shock) reliably leads to alterations in non-emotional temporal perception (Sarigiannidis et al. 2020a). We found clear evidence that anxiety leads to underestimation of time, i.e. that time ‘speeds up’ under threat of unpredictable electric shock, possibly due to the loss of temporal information (Sarigiannidis et al. 2020a, 2020b).

We previously interpreted this underestimation as reflecting a loss of temporal information under anxiety, potentially via attentional resource competition. However, in Sarigiannidis et al. (2020b) we found that a purely cognitive load manipulation, while taxing attention, did not produce temporal underestimation. This suggests that the mechanism underlying anxiety-related temporal distortion may not reduce to simple attentional depletion and may instead involve wider affective–cognitive neural systems. This motivated the present study’s focus on identifying whether anxiety and temporal processing rely on shared neural substrates, using a similar time estimation task.

Previous functional magnetic resonance imaging (fMRI) studies employing a similar anxiety manipulations to ours (McMenamin et al. 2014, Kirlic et al. 2017) have consistently found activation in the anterior insula while participants passively anticipate unpredictable shocks (Robinson et al. 2019). Other brain areas consistently activated during sustained threat include the cingulate gyrus, thalamus, caudate and cerebellum (Mechias et al. 2010, Robinson et al. 2019). These brain areas have also been associated with processing and anticipating painful stimuli (Koyama et al. 2005, Wager et al. 2013), supporting the hypothesis that they may play a general role in prolonged states of negative anticipation. Consequently, we would expect to replicate these activations in the present study when individuals are under threat-of-shock-induced anxiety. Given this consistent involvement of cingulate subregions in anticipatory anxiety, and preliminary evidence from Study 1 (presented in the Supplementary Material), we considered the cingulate cortex a candidate region for anxiety-related activation in our confirmatory Study 2.

A broad network of brain regions has been reported to be recruited during time perception. A recent study suggested that similarly to sensory cortical maps, topographic timing maps exist; where different brain areas respond to specific ranges of temporal intervals, and whose selectivity changes gradually (Harvey et al. 2020). For supra-second intervals (the focus of the current work), cortical brain regions are more heavily involved (Wiener et al. 2010, Nani et al. 2019), including the cingulate and frontal cortex, as well as the pre-supplementary motor area (pre-SMA) which is considered central (Schwartze et al. 2012). Recent meta-analytic work (Mondok and Wiener 2023) further highlights the involvement of cingulate, insular, and prefrontal regions during supra-second timing.

No prior studies have explored the interaction between anxiety and time perception at the neural level. However, more broadly, previous studies have highlighted the involvement of frontal areas in the interaction between anxiety and non-affective cognitive tasks (Bishop 2007, 2009, Carlisi and Robinson 2018, Robinson et al. 2019). Consistent with this, two prior threat-of-shock studies found that anxiety increased activation in frontal areas (including the superior frontal gyrus), and these activations were also associated with anxiety-related behavioural change (Torrisi et al. 2016, Balderston et al. 2017). However, assuming that the effect of anxiety on cognitive function can be likened to classic multitasking interference (where two tasks compete for limited resources, and hence interfere with one another: Eysenck et al. 2007, Watanabe and Funahashi 2018), the precise region(s) implicated may be where overlapping resources are engaged (Nijboer et al. 2014, Watanabe and Funahashi 2018, Maillet et al. 2019). We have previously argued that the impact of anxiety on time perception is driven by demands on attention, a cognitive resource both of these processes might be utilizing (Sarigiannidis et al. 2020a, 2020b). Thus, it is possible that the impact of induced anxiety on temporal perception is driven by overlap between time- and anxiety-related neural processing. A strong candidate brain area for such overlap is the pre-SMA. Time-perception related activations in the pre-SMA have been reported to vary parametrically with the amount of attention allocated to timing a stimulus (Coull et al. 2004). At the same time, the pre-SMA seems to be activated by threat-of-shock manipulations as revealed by a meta-analysis (Chavanne-Arod and Robinson 2020).

To test our hypothesis, we initially conducted a small exploratory study (Study 1), which was used to refine our design and generate pre-registered (Sarigiannidis 2019) predictions and an analysis plan for the second study (Study 2). Importantly, for Study 2, we calibrated our temporal cognition task to each participant to exclude the possibility that any neural differences observed were due to the properties of the different temporal intervals of the task. Our specific predictions for Study 2, were the following:

1. Induced anxiety would lead to temporal underestimation, replicating our previous finding (Sarigiannidis et al. 2020a). Specifically, we predicted that participants would perceive the temporal intervals as shorter when under threat of shock.
2. Anxiety would elicit activation in the cingulate cortex and the caudate, as suggested by results from our (pilot) Study 1 (presented in the Supplementary Material and our pre-registration).
3. Time perception would elicit activation in the pre-SMA and right inferior frontal gyrus, as identified in a previous meta-analysis (Wiener et al. 2010).
4. Time-perception-related and anxiety-related neural processing would interact in the pre-SMA

## Materials and Methods

### Overview

All studies consisted of a single testing session ( Table 1). Following written informed consent, as approved by local ethical procedures (see below for specifics), and the completion of questionnaires, a shock calibration procedure was completed by the participant in the scanning room to determine an appropriate level of aversive electrical stimulation. Participants then completed the temporal bisection task under threat-of-unpredictable-shock and safe conditions inside the scanner. During each one-hour scan, anatomical and functional images were acquired, with each of the two functional runs lasting approximately 15 minutes. During the task, participants selected between two responses (short and long) in a two-alternative forced choice manner inside the scanner, via an MRI-compatible button box. Study 1 was an exploratory pilot used to generate regions of interest for Study 2.

### Study site

Study 1 was completed at the National Institutes of Health, Bethesda, MD, USA, while Study 2 was completed at University College London (UCL), UK.

### Apparatus

In Study 1, experiment material was presented on Windows computers using E-prime, while Study 2 was run in Cogent 2000 (www.vislab.ucl.ac.uk/cogent.php; Wellcome Trust Centre for Neuroimaging and Institute of Cognitive Neuroscience, UCL, London), under Matlab.

### Shock calibration

A shock calibration procedure was performed prior to testing in order to control for shock tolerance and skin resistance. Single pulse shocks (Study 1) or trains of shocks (Study 2; as these are more aversive and permitted by the equipment at this site) were delivered to the non-dominant wrist via a pair of silver chloride electrodes using a DS7 (Study 1) or a DS5 (Study 2) stimulator (Digitimer Ltd, Welwyn Garden City, UK). Participants received shocks sequentially with stepped increases in amplitude, which they rated using a scale from 1 to 10 (1 labelled “I barely felt it” and 10 labelled “approaching unbearable”). The level of shock delivered in the experiment was set to 80% of the maximum tolerated for each individual.

### Temporal bisection task under threat of shock: overview

**Figure 1.**
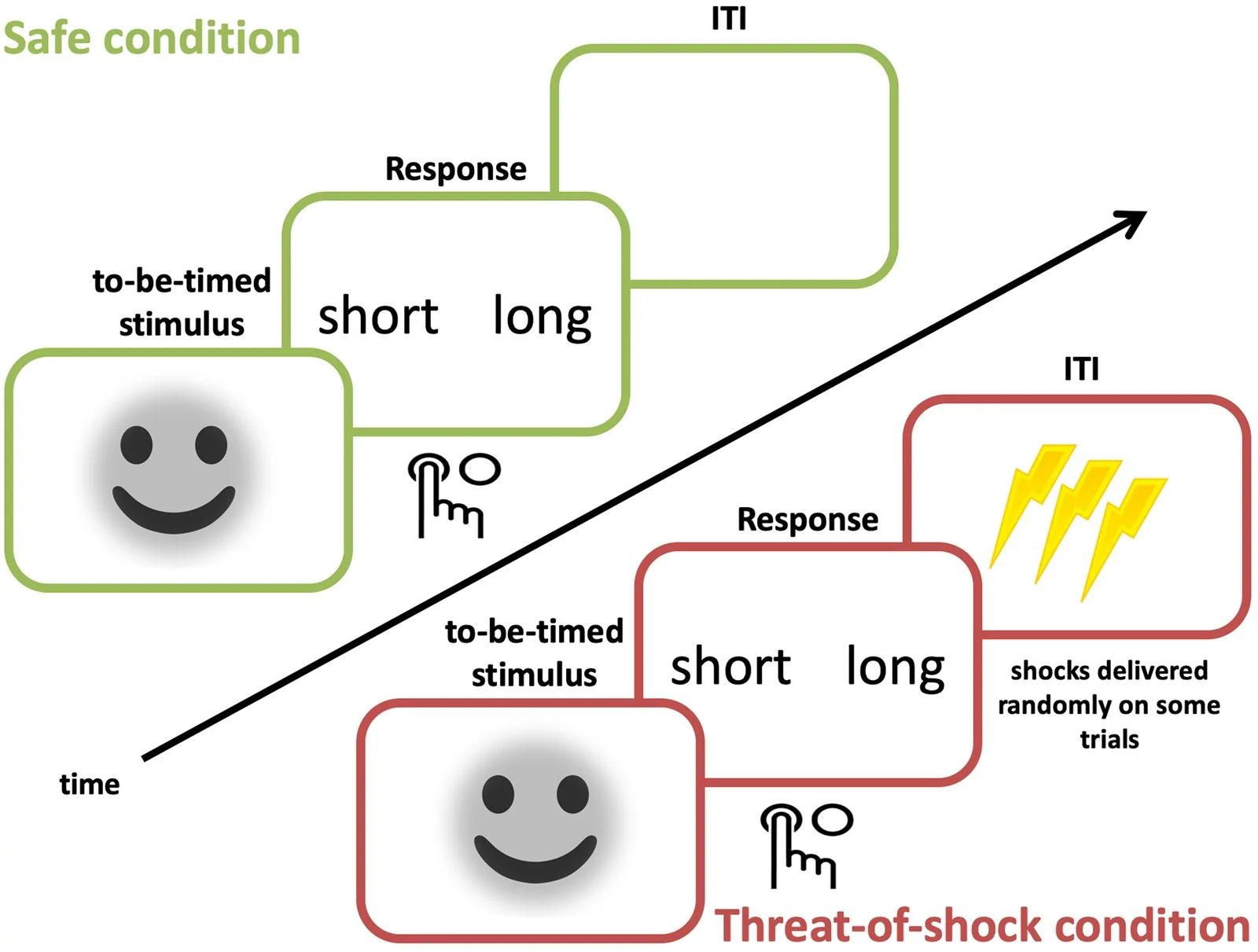
Experimental parameters for the studies and demographic information. Stimulus durations differed between Studies 1 and 2 (see “Experiment specific methods” section). Participants were given 1.5 s to respond and the inter-trial intervals (ITIs) were 0.5, 1.1, 1.7, 2.3, 2.9, and 3.5 s. Note: In the actual experiment, participants were presented with NimStim images (Tottenham et al. 2009): colour photographs of actors producing emotional expressions.

In both Study 1 and 2, participants completed a visual temporal bisection task under two alternating conditions (Fig. 1): “threat-of-shock” (labelled “threat”), during which they could receive shocks at any time without warning; and “safe”, during which they could not receive any shocks (the order was counterbalanced). The task was flanked by coloured borders that indicated the condition (safe or threat), taken from a pool of four colours (red, blue, green, magenta), which was also counterbalanced across participants.

A short training phrase preceded the main task. This involved presenting participants with two anchor durations (Fig. 1), a “short” duration (1.4 s) and a “long” duration (2.6 s). Each was shown three times, and presentation order was pseudorandomized. In addition, before the beginning of each block (safe or shock) the anchor durations were repeated to ensure consolidation.

Consistent with our previous studies (Sarigiannidis et al. 2020a, 2020b), the to-be-timed stimuli were pictures of emotional facial expressions (happy, fearful or neutral; taken from a standardized set: Tottenham et al. 2009). Stimulus durations differed between Study 1 and 2 (see below, Experiment specific methods). All stimulus types and durations were pseudorandomized, and presented equally often in each threat and safe block to avoid potential biases (Wearden and Ferrara 1996). On each trial participants were required to: press “short” if the duration of the stimulus was more similar to the “short” anchor, or press “long” if the duration of the stimulus was more similar to the “long” anchor (left and right buttons for these options were counterbalanced across participants). After the 1.5 s response limit, there was a variable inter-trial interval (ITI: 0.5, 1.1, 1.7, 2.3, 2.9, and 3.5 s, pseudo-randomized). Participants were explicitly told to avoid counting seconds as well as avoid any other strategy to estimate the duration of the stimuli; instead they were instructed to make the temporal judgments based on their gut feeling.

### Psychophysical modelling

We fitted psychometric functions to each participant’s data, separately for the safe and the threat condition in order to calculate the bisection point (BP). Briefly, the BP represents the duration that seems equally ‘short’ and ‘long’ to a participant. Lower BP values indicate temporal overestimation, whereas higher BP values indicate temporal underestimation, as described in our previous papers (Sarigiannidis et al. 2020a, 2020b). Psychometric functions were fitted using the Palamedes toolbox in MATLAB (Prins and Kingdom 2009), and goodness-of-fit was visually inspected for each participant.

## Experiment specific methods

Supplementary Material provides full details of participant recruitment and screening, ethical approvals, task designs for Studies 1 and 2, shock administration protocols, MRI acquisition parameters, and behavioural data analysis procedures. Additional notes are included on data exclusions, reporting transparency, and corrections to the pre-registration record.

### Functional neuroimaging data analysis

Study 1 was a pilot study which we used to generate ROIs for Study 2, and hence the data analysis for Study 1 was exploratory. In both studies, EPI data were analysed using Statistical Parametric Mapping 12 (SPM12; Wellcome Trust Centre for Neuroimaging, London, www.fil.ion.uck.ac.uk/spm) in Matlab R2015b. After removing the first five volumes (dummy scans) from each time series to allow for T1 equilibration, the remaining volumes were realigned to the sixth volume, normalized into standardized space [Montreal Neurological Institute (MNI) template], and smoothed using an 8 mm full-width-at-half-maximum Gaussian kernel. Following the realignment stage, all image sequences were checked for translations and rotations greater than 1.5 mm/1 degree, and corrupted images were removed and replaced using interpolation. Following spatial normalization, images were manually checked for artefacts. Brain areas reported were defined using the Atlas of the Human Brain (Mai 2016). Details of the functional neuroimaging data analysis for Study 1 are provided in the Supplementary Material.

#### Study 2

Due to changes in the experimental design, we modelled the entire trial including stimulus presentation, stimulus response and ITI. Our four regressors of interest were threat trials (BP trials during the threat condition) and safe trials (BP trials during the safe condition), which were categorized as either “perceived long” (BP trials on which the participant responded with “long”) or “perceived short” (BP trials on which the participant responded with “short”). Regressors of no interest were the catch trials (i.e. trials whose duration was 1.4 and 2.6 s) as well as missed trials (i.e. trials on which participant did not make a response), which were modelled separately for safe or threat blocks. Other regressors of no interest were training stimuli indicating the anchor durations (presented before the beginning of each block), shocks, as well as the start screens of each block (indicating whether participants will be safe or under threat of shock). All these regressors were convolved with SPM’s canonical hemodynamic response function time-locked to the onset of the corresponding event, and considering its duration (which varied slightly across participants due to variation in BPs).

We also included six movement regressors of no interest in all participants, alongside 12 regressors extracted from the pulse and respiratory rate, corresponding to a set of sine and cosine Fourier series components extending to the third harmonic (Glover et al. 2000) based on traces produced by the Spike software. There were additionally two regressors to model the variation in respiratory volume (Birn et al. 2006, 2008) and heart rate (Chang et al. 2009), also based on the spike traces.

Using the general linear model, parameter estimate images were created for each regressor, and combined to create the primary contrasts at the subject level.

Second-level analyses were conducted using the standard summary statistics approach to random effects analysis. We applied a cluster-forming threshold of *P* < .005 (uncorrected) and report small-volume corrected p-values for responses in our ROIs as defined in our pre-registration document (https://osf.io/54qfh).

The fMRI contrasts were: (i) the effect of threat, i.e. all the BP trials under the threat-of-shock condition, compared to the safe condition; (ii) perceived duration (“long” vs “short”, though the actual duration was identical), including all the BP trials, collapsed across the threat and safe conditions; (iii) the interaction between perceived duration and threat. We additionally examined the overlap between (i) and (ii) using conjunction analysis.

#### Regions of interest

In Study 1 we detected significant threat-induced activation in the cingulate cortex, and since this area was also activated during threat-of-shock conditions in our group’s previous studies (Robinson et al. 2014) we used it as a pre-registered ROI. However, due to an error the co-ordinates identified as the cingulate cortex in the pre-registration document actually refer to the left caudate, because both peaks fell within the same large cluster. In the interest of full transparency, we therefore used both ROIs for the threat contrast using a 10-mm diameter sphere for both the caudate (MNI coordinates [*x*=−18, *y* = 11, *z* = 26]) and the mid-cingulate cortex (MNI coordinates [*x* = 0, *y*=−4, *z* = 50]; prediction 2). We used spheres centred on these peaks for simplicity, which avoided the need to decide where to divide the large cluster observed in Study 1.

A previous meta-analysis on time perception studies reported strong activation in the pre-SMA (Wiener et al. 2010), and thus for contrast (1) we defined an additional ROI as a 10-mm sphere ROI on that area (again this was pre-registered: Talairach coordinates [*x* = 0, *y* = 0, *z* = 56], taken from (Wiener et al. 2010), converted to MNI coordinates [*x*=−1, *y*=−4, *z* = 62]; prediction 3).

## Results

### Study 1 (see the Supplementary Material) identified threat-related activation peaks in the cingulate cortex and caudate, which were then pre-registered as regions of interest for study 2

#### Behavioural results inside the scanner

**Figure 2.**
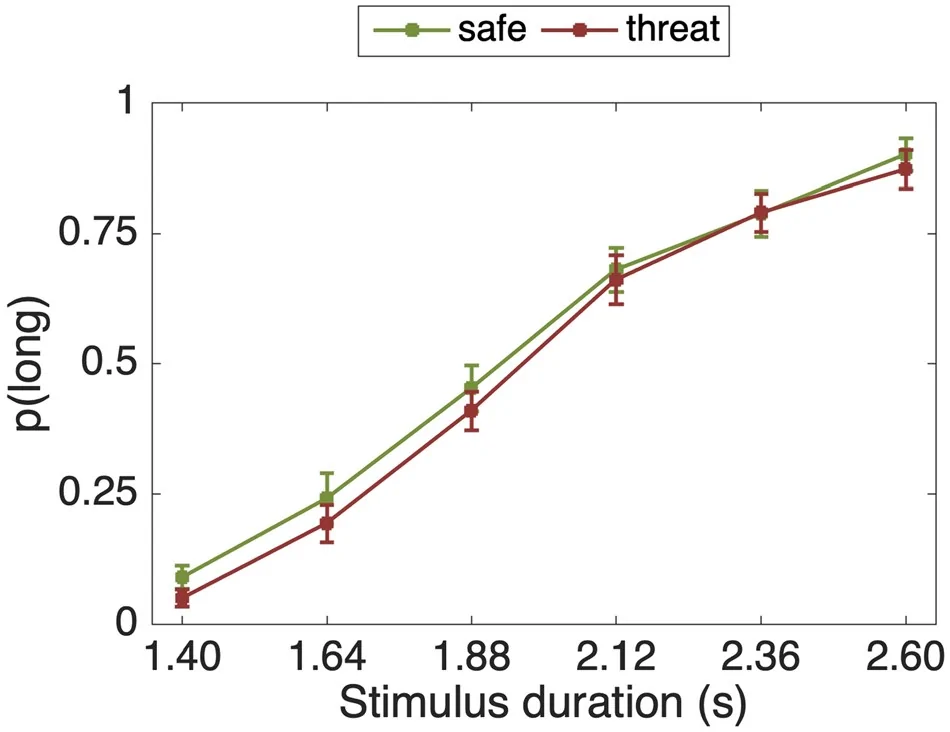
Proportion of stimuli rated “long” as a function of the actual presentation length and threat condition. Error bars are standard errors of the mean (SEM).

Psychometric functions for all stimulus durations are shown in Fig. 2 (included for comparison with our previous work). However, in Study 2 the behavioural analyses focused exclusively on BP trials, as the remaining durations were used solely for task calibration and were not included in inferential analyses.

**Figure 3.**
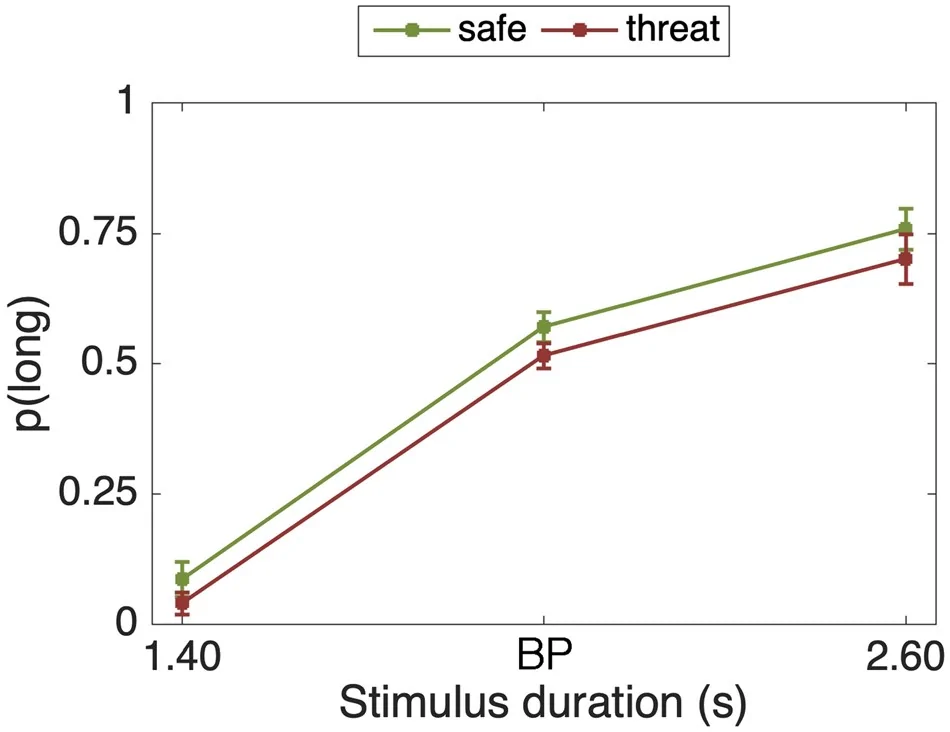
The proportion of *P*(long) responses was significantly lower in the threat condition for the individually tailored bisection point (BP) duration, suggesting temporal underestimation during anxiety. Error bars are standard errors of the mean (SEM). The 1.4 and 2.6 s durations were not used in any analyses and are only plotted here for completeness.

Participants reported being significantly more anxious in the threat compared to the safe condition (*t*(28)=11.28, *P* < .001, *d* = 2.09). As hypothesized, on BP trials participants responded “short” significantly more often in the threat compared to the safe condition (*t*(28)=2.39, *P* = .024, *d* = 0.44; Fig. 3).

#### Neural effect of threat

##### Threat > safe

**Figure 4.**
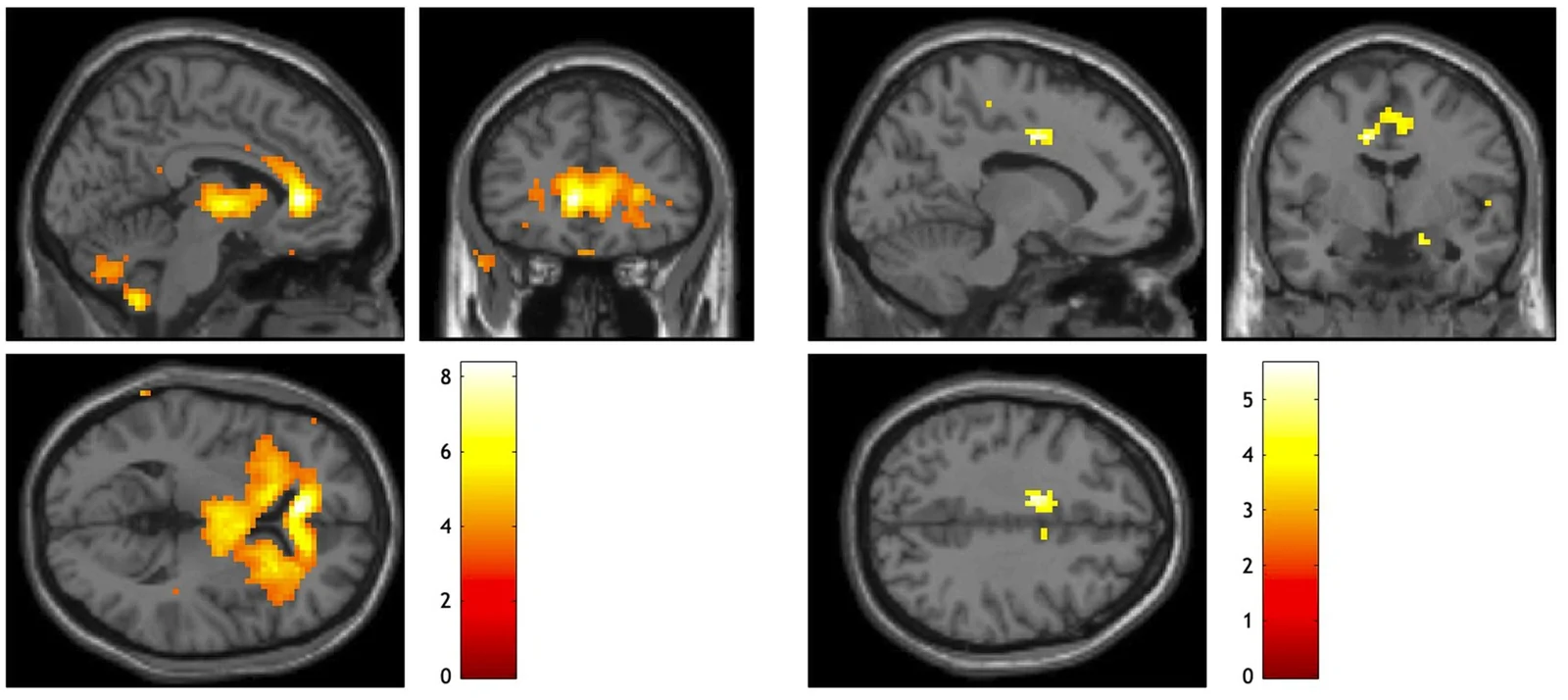
Activation for the threat > safe contrast (left) and the perceived long > perceived short trials (right). Cluster forming threshold *P* < .005 (uncorrected). The colour bar represents *t*-values.

This analysis examined the effect of the threat-of-shock vs the safe condition. There was significant (whole-brain voxel-level FWE corrected) activation in a large cluster (see Fig. 4 and Table 2), including peaks in the subgenual anterior cingulate cortex (sgACC, bilateral), thalamus (bilateral), claustrum (left only), caudate (left only), and anterior insula (bilateral). Other peaks in this cluster that did not survive voxel-level correction were an insula/orbitofrontal cortex area (right only), the lateral septal area (right only), and the putamen (right only). Three more significant clusters revealed activations in the left cerebellum and the parietal operculum (left and right).

**Table 1.** Summary of studies conducted.

	Sample size	Stimulus duration	Shock type	BDI	STAI
Study 1: NIH	13	1.4, 1.64, 1.88, 2.12, 2.36, 2.6 s	Single pulse	n/a	n/a
Study 2: UCL	29	BP (78% trials of interest), 1.4 s and 2.6 s (22% catch trials)	Train of shocks (0.5 s)	5.06 (5.53)	35.72 (12.48)

Note that the studies were completed at different sites in different countries. Note also that Study 2 attempts to address a confound in Study 1 (i.e. that observed activations could be driven by in differences in the durations of the stimuli themselves). Hence, in Study 2 participants only saw a single intermediate stimulus set to their own bisection point, with the extremes presented only 22% of the time.

**Table 2.** fMRI activations for threat > safe, safe > threat, perceived long > perceived short, and perceived short > perceived long contrasts (cluster forming threshold: *P* < .005, uncorrected).

			MNI coordinates					Cluster	Peak
	Region	Hemisphere	*x*	*y*	*z*	#voxels	Z-value	*P*(FWE-corr)	*P*(FWE-corr)
**Threat > safe**	Subgenual anterior cingulate cortex	Left	−6	32	5	4951	5.87	<.001	0
	Thalamus (inferior thalamic peduncle)	Left	−6	−7	2	“	5.2	“	.004
	Claustrum	Left	−24	20	−1	“	4.95	“	.012
	Subgenual anterior cingulate cortex (BA33)	Right	9	32	5	“	4.92	“	.013
	White matter (inferior longitudinal fasciculus)	Right	33	2	−10	“	4.86	“	.017
	Thalamus (posterior hypothalamic area)	Right	3	−10	2	“	4.86	“	.017
	Caudate	Left	−15	17	8	“	4.74	“	.027
	Anterior insula	Right	30	14	−10	“	4.66	“	.038
	Anterior insula	Left	−30	14	−7	“	4.58	“	.051
	Insula/orbitofrontal cortex (area orbitoinsularis)	Right	27	20	5	“	4.52	“	.064
	Lateral septal	Right	3	2	5	“	4.49	“	.071
	Putamen	Right	15	8	−1	“	4.35	“	.118
	Putamen	Right	18	14	2	“	4.31	“	.137
	White matter	Right	24	26	−4	“	4.3	“	.14
	Putamen	Right	18	20	2	“	4.25	“	.165
	White matter	Right	27	32	8	“	4.25	“	.165
								
	Cerebellum	Left	−9	−55	−52	1914	4.97	<.001	.01
								
	Parietal operculum	Left	−69	−25	26	278	4.06	.042	.295
								
	Parietal operculum	Right	45	−46	29	364	3.82	.013	.528

			MNI coordinates					Cluster	Peak
	Region	Hemisphere	x	y	z	#voxels	Z-value	*P*(FWE-corr)	*P*(FWE-corr)
**Safe > threat**	Inferior temporal gyrus	Left	−36	5	−40	266	5.4	.05	.002
	Inferior temporal gyrus	Left	−33	17	−37	“	5	“	.009
	Inferior temporal gyrus	Left	−30	14	−34	“	4.82	“	.02
	Medial orbital gyrus	Left	−21	32	−19	“	4.56	“	.055
	Medial orbital gyrus	Left	−24	20	−22	“	4.37	“	.108
	Medial orbital gyrus	Left	−18	29	−16	“	4.23	“	.173
	Amygdala (lateral amygdaloid)	Left	−24	−1	−28	“	3.7	“	.662
	Amygdala (basolateral amygdaloid)	Left	−21	−4	−25	“	3.54	“	.818
	Presubiculum	Left	−18	−13	−25	“	3.5	“	.857
								
	Parahippocampal (subiculum)	Right	18	−10	−25	262	4.42	.053	.093
	Inferior temporal gyrus	Right	30	14	−34	“	4.34	“	.121
	Inferior temporal gyrus	Right	36	8	−40	“	4.15	“	.225
	inferior temporal gyrus	Right	42	17	−43	“	4.1	“	.259
	White matter	Right	33	−1	−34	“	4.01	“	.335
	White matter	Right	27	2	−31	“	3.82	“	.532
	Amygdala (lateral amygdaloid)	Right	24	−1	−28	“	3.66	“	.7

			MNI coordinates					Cluster	Peak
	Region	Hemisphere	*x*	*y*	*z*	#voxels	Z-value	*P*(FWE-corr)	*P*(FWE-corr)
**Long > short**	White matter	Left	−12	−4	38	93	4.58	.016	.077
	Mid cingulate	Right	6	−1	44	“	3.79	“	.72
	Mid cingulate	Left	0	−4	50	“	3.46	“	.963

			MNI coordinates					Cluster	Peak
	Region	Hemisphere	*x*	*y*	*z*	#voxels	Z-value	*P*(FWE-corr)	*P*(FWE-corr)
**Short > long**	Cerebellum	Right	27	−73	−46	303	4.33	.148	.184
	Inferior temporal gyrus	Left	−51	−46	−10	107	4.15	.187	.321
	Cerebellum	Left	−18	−40	−46	38	4.14	.276	.331

We defined the caudate and the mid-cingulate cortex as ROIs, since they were both activated under threat-of-shock in Study 1. When small volume correction (SVC) was applied using a 10-mm sphere ROI around the peak of the caudate cluster identified in Study 1 (MNI coordinates [*x*=−18, *y* = 11, *z* = 26]), a peak survived FWE voxel-level correction for multiple comparisons ([*x*=−15, *y* = 20, *z* = 23], *Z* = 3.21, *k* = 37, *P*_SVC_ < .05). The ROI around the peak of the mid-cingulate cortex cluster from Study 1(MNI coordinates [*x* = 0, *y*=−4, *z* = 50]), also revealed a peak surviving FWE voxel-level correction for multiple comparisons ([*x* = 3, *y*=−4, *z* = 41], *Z* = 2.90, *k* = 11, *P*_SVC_ < .05).

To explore brain-behaviour correlations, we performed an exploratory analysis in which the effect of threat on behavioural responses (p(Long)_threat_ − p(Long)_safe_) was entered as a covariate into the [threat>safe] contrast. We expected that the neural effect of threat>safe would be larger in participants who showed greater temporal underestimation during threat. However, no activations survived correction for multiple comparisons. Equally, no activations survived correction in the inverse contrast.

##### Safe > threat

This analysis examined the effect of the safe vs the threat-of-shock condition. There was significant (whole-brain voxel-level FWE corrected) activation in two clusters with bilateral peaks in the inferior temporal gyrus, in the left medial orbital gyrus (see Fig. 4 and Table 2), and in a right parahippocampal area (subiculum). Both clusters extended into the amygdalae, although the peaks there did not survive voxel-level correction.

#### Neural effect of perceived duration

##### Perceived long > perceived short trials

This analysis examined the effect of the perceived “long” vs. “short” trials, i.e. based on the participants’ judgments of the to-be-timed stimulus (which was actually always the same duration, at their own BP). There was significant activation in a single cluster with bilateral peaks in mid-cingulate cortex (see Fig. 4 and Table 2).

In our pre-registration document, we defined the supplementary motor area as an ROI since it has been reliably implicated in time perception studies. When small volume correction was applied using a 10-mm sphere ROI around the meta-analytic peak identified by Wiener et al. (2010: MNI coordinates [*x*=−1, *y*=−4, *z* = 62]) we identified a mid-cingulate cortex peak which survived FWE voxel-level correction for multiple comparisons ([*x* = 0, *y*=−4, *z* = 53], *Z* = 3.14, *k* = 11, *P*_SVC_ < .05).

Additionally, in an exploratory analysis, we investigated whether activation in the mid-cingulate cortex peak voxel was associated with the degree of temporal underestimation during threat, but the correlation was non-significant (*r*(29)=−.28, *P*=.884).

##### Perceived short > perceived long trials

This analysis examined the effect of the perceived short vs. long trials, i.e. trials in which participants judged the to-be-timed stimuli as “short,” compared to when they judged them as “long.” No clusters survived correction at either the peak or voxel level (Table 2).

#### Neural effect of threat × perceived duration

This analysis examined the interaction between the effect of threat and that of perceived duration. No clusters survived correction in either this or the inverse contrast.

#### Overlap analyses

**Figure 5.**
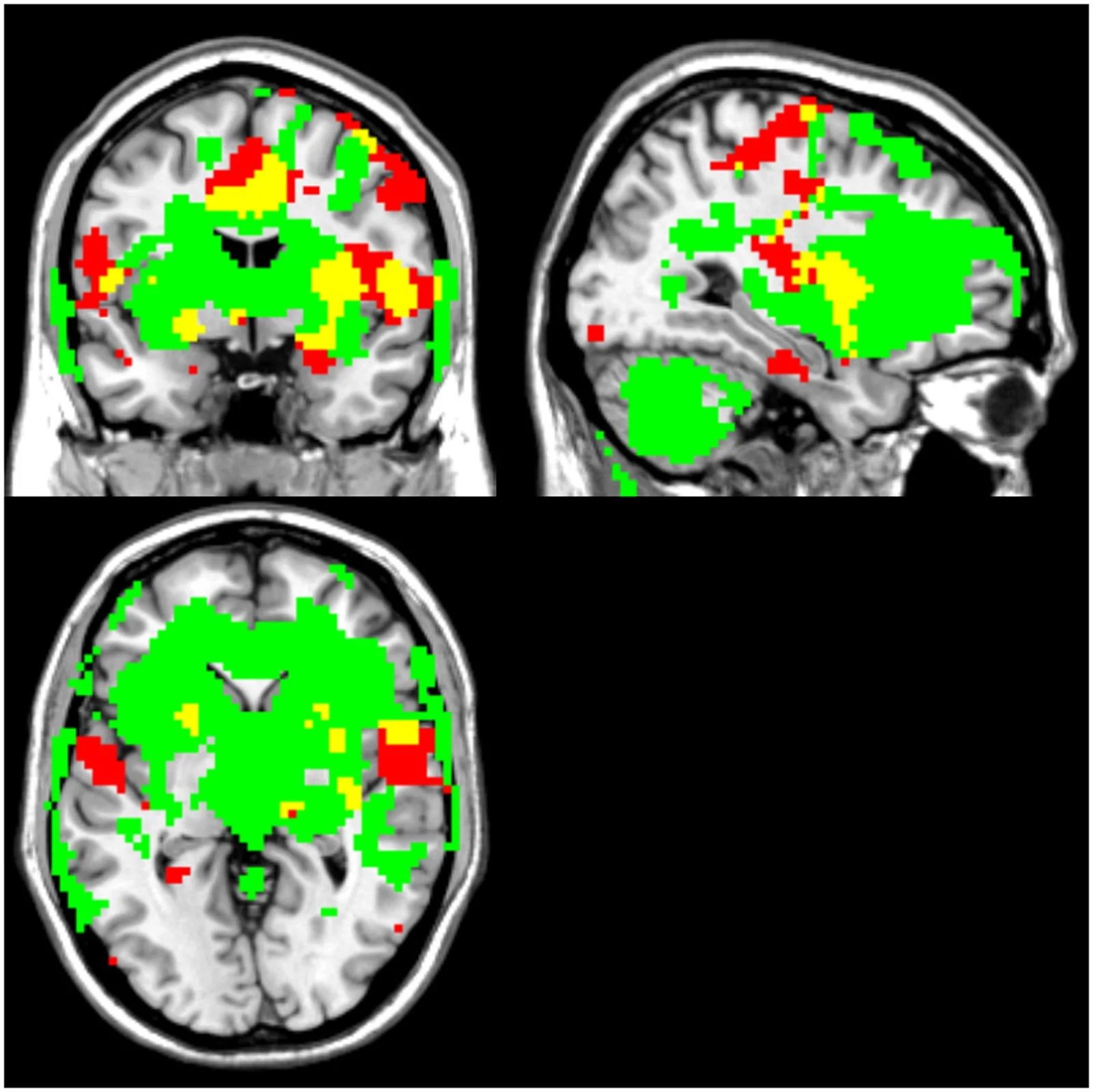
Overlapping activations of threat > safe (in green) and perceived long > perceived short contrasts (in red). Overlapping regions (in yellow) include the insula ([*x*=−27, *y* = 8, *z*=−7]), putamen ([*x* = 30, *y* = 2, *z*=−7]), and mid-cingulate ([*x*=−12, y = 2, *z* = 38]). Figure generated by creating masks from the threat > short and perceived long > perceived short contrasts, both thresholded at *P* < .05 (uncorrected) for display purposes Note: Activation of other brain areas has been omitted for clarity.

The results of Study 2 suggest a degree of overlap in the activations identified in the [threat>safe] and [perceived long>perceived short] contrasts, specifically in the insula, putamen, and mid-cingulate cortex. We formally tested this overlap by creating a mask for each contrast (thresholded at *t* > 1.7, corresponding to *P* < .05 uncorrected), using this to perform small volume correction on the other contrast (see Fig. 5), using a cluster-forming threshold of *p* < .005 (uncorrected).

Applying the [perceived long>perceived short] mask to the [threat>safe] contrast revealed overlapping clusters surviving FWE voxel-level correction for multiple comparisons in right insula ([*x* = 30, *y* = 2, *z*=−7], *Z* = 4.35, *k* = 57, *P* < .05) and left putamen ([*x*=−27, *y* = 8, *z*=−7], *Z* = 4.03, *k* = 12, *P* < 0.05). This indicates these areas are recruited both during threat anticipation and when participants perceive a stimulus as longer.

Applying the [threat>safe] mask to the [perceived long>perceived short] contrast revealed overlap in a mid-cingulate cortex area which narrowly missed FWE voxel-level correction for multiple comparisons ([*x*=−12, *y* = 2, *z* = 38], *Z* = 4.29, *k* = 15, *P* = .068). This suggests that mid-cingulate cortex are involved in both anxiety-related and timing-related processes.

## Discussion

A key aim of this study was to test the proposal that anxiety-induced temporal underestimation reflects competition for shared neural resources. The overloading account predicts that if threat and temporal estimation draw upon overlapping neural substrates, then increased threat-related demand should limit the resources available for accurate temporal encoding, producing temporal underestimation. We therefore explored the neural correlates of anxiety-induced temporal speeding by combining a threat-of-shock manipulation and a temporal bisection task.

We replicated and extended our previous behavioural findings (Sarigiannidis et al. 2020a) in the scanner, showing that participants underestimated the duration of a single temporal interval (corresponding to their BP) when anxious (prediction 1). We further found that induced anxiety activates the ACC and caudate (prediction 2), as well as the insula, consistent with previous studies (McMenamin et al. 2014, Balderston et al. 2017, Torrisi et al. 2016, 2018, Meyer et al. 2019, Robinson et al. 2014, 2019).

Additionally, although the perception of longer temporal intervals was associated with activation in the pre-SMA (prediction 3) consistent with previous studies (Wiener et al. 2010, Schwartze et al. 2012) this region did not emerge in the whole brain analysis. Instead, a mid-cingulate area was more robustly activated when participants perceived the temporal interval as long.

Finally, consistent with our “overloading” hypothesis, activations in the threat and perceived duration contrasts overlapped in the insula and mid-cingulate area (but not in the pre-SMA; prediction 4). This convergence indicates that both functions rely on partially shared neural resources. Such anatomical overlap provides the necessary condition for resource competition: when threat recruits these regions, less capacity remains for precise temporal encoding, leading to the observed temporal underestimation.

### Neural correlates of anxiety induced by threat of shock

The pattern of anxiety-induced neural activation (threat>safe) was largely consistent with previous studies. Specifically, the whole brain analysis in Study 2 revealed a large cluster of activation in the ACC, with a peak in the sgACC. This cluster included the caudate and a mid-cingulate area identified in Study 1, which we confirmed using small-volume correction, confirming prediction 2.

Since our participants received more electrical shocks than in previous threat-of-shock studies (e.g. Robinson et al. 2014, Torrisi et al. 2016, Balderston et al. 2017), it is possible that the sgACC activation is due to processing painful stimuli (for a review see Palomero-Gallagher et al. 2015). However, we did regress out the effect of shocks, hence activation in this region is most likely due to shock anticipation. This is consistent with sgACC activation being considered central in sustained anticipatory responses in both primates and humans (Robinson et al. 2014, Rudebeck et al. 2014, Wallis et al. 2017).

At the same time, the right insula and the right caudate were activated across Study 1 and 2. These areas have previously been implicated in induced anxiety (insula: McMenamin et al. 2014, Balderston et al. 2017, Meyer et al. 2019); caudate: McMenamin et al. 2014, Torrisi et al. 2016). Insula is often co-activated with the ACC (Palomero-Gallagher et al. 2015) and is considered to be part of a putative “anxious anticipation” network (McMenamin et al. 2014). Although the caudate is less consistently implicated in threat-of-shock studies, a previous study has similarly found activation in the right caudate (Torrisi et al. 2016). Considering that the caudate is considered key area in pathological anxiety, and is a target for deep brain stimulation of disorders in which anxiety plays a core role (i.e. obsessive compulsive disorder; Alonso et al. 2015), future studies could further explore how it relates to anticipatory anxiety. Taken together, these results highlight important roles of sgACC, the insula and the caudate in anticipatory anxiety.

### Neural correlates of temporal perception

Our results suggest that a mid-cingulate area was more active when participants perceived a stimulus as “long” than when they perceived the exact same stimulus as “short.” It is possible that this mid-cingulate area is involved in monitoring stimulus duration, where increased activation reflects more efficient processing (i.e. fewer temporal pulses were lost). The cingulate cortex has previously been implicated in time perception (for a recent meta-analysis see Nani et al. 2019), but less consistently than other brain areas such as the pre-SMA. This apparent discrepancy might be attributed to the different experimental tasks used. Specifically, previous fMRI studies on perceptual timing have mainly employed comparative temporal discrimination tasks, in which participants judge which of the two consecutively presented temporal intervals was longer. Thus, in these studies the neural signal may represent general perceptual timing, including processes such as keeping track of different temporal intervals and working memory; since durations have to be kept in mind to allow comparisons on each trial. In our task, participants viewed the exact same temporal interval which they compared with temporal durations they had consolidated (i.e. the anchor durations); hence the neural signal reflects differences in perception free of working memory confounds or any other confounds related to stimulus duration.

Nevertheless, the pre-SMA has been implicated across different temporal cognition tasks, from motor (e.g. finger tapping) to perceptual and is considered a key area in timing (Wiener et al. 2010, Schwartze et al. 2012, Nani et al. 2019). In our study, pre-SMA activations did not survive correction in the whole-brain analysis, which suggests that other regions may be more important in our study. This raises questions about precisely what role the pre-SMA plays in keeping track of time. It is also possible that the pre-SMA participates in some general aspect of temporal processing, such as using strategies to count interval durations, which might explain why it is so ubiquitously activated across so many different temporal cognition tasks (Nani et al. 2019).

Finally, we found overlapping mid-cingulate cortex activation in the threat and perceived duration contrasts. This convergence raises the possibility that mid-cingulate cortex might be implicated in emotion-related alterations in temporal perception, in-line with the hypothesized role of this region in mediating cognitive affective and behavioural responses to anxiety (Grupe and Nitschke 2013).

### Neurocognitive mechanisms of temporal underestimation under anxiety

We previously hypothesized that the effect of anxiety on temporal cognition was due to dual task interference: anxiety may occupy limited neurocognitive resources, thus altering performance in the temporal estimation task (Sarigiannidis et al. 2020a, 2020b). Specifically, in this study we considered the threat-of-shock condition to represent a dual-task scenario, since participants are performing the temporal task whilst also “processing” anxiety, and the safe condition to be single-task, since participants are only performing the temporal task.

Notably, the dual-task load manipulation in Sarigiannidis et al. (2020b) did not induce temporal compression despite taxing attention, suggesting that anxiety-related temporal distortion cannot be attributed solely to generic attentional depletion. The present work extends this by showing that anxiety recruits mid-cingulate regions also implicated in timing, providing a more specific candidate mechanism.

We found preliminary evidence that insula and a mid-cingulate area were activated both during the threat and temporal contrasts. It is thus possible that anxiety-related insula activity (Simmons et al. 2006, Baur et al. 2013) interfered with the mid-cingulate cortex (an area associated with time perception Nani et al. 2019), leading it to accumulate temporal information less efficiently (e.g. losing ‘temporal pulses’) and thus resulting in the temporal underestimation we observed. However, this hypothesis is not completely supported by our data, considering that we did not find a significant threat-by-perceived duration interaction either at the whole-brain level, or when specifically examining the insula or mid-cingulate (see the Supplementary Material). In fact, one might expect no interaction between the insula and mid-cingulate activation in the interaction contrast in participants without threat-induced time underestimation; but an interaction (due to overloading) in those who did underestimate time under threat. However, our data did not support this either: we did not detect any correlation across participants between the underestimation of time during threat and either insula or mid-cingulate activation in the interaction contrast. Future work could explore this hypothesis including more participants to provide greater power to detect individual differences (rather than just within-subject differences) and by employing a similar task with a fully factorial design, additionally incorporating a control/passive task. This might provide a more complete picture of how anxiety and cognition interact at the neural level.

Previous studies have suggested that the dorsolateral prefrontal cortex is implicated in the cognitive alterations due to trait (Bishop 2009) and induced anxiety (Torrisi et al. 2016, Balderston et al. 2017). However, we did not find such activation here. This discrepancy can be explained considering that the aforementioned studies used cognitively demanding, fast-paced tasks which are more likely to activate prefrontal areas (Höller-Wallscheid et al. 2017) than our simple task. Taken together our results tentatively support the idea of anxiety altering cognition similarly to dual-task situations, but in our data there was no evidence that this was mediated by dorsolateral prefrontal activation.

### Limitations

Study 1 was a pilot study with a different design to Study 2. In Study 1 participants had to judge the duration of six different temporal intervals. This version of the task confounded duration and time perception, such that effects might simply be driven by the duration of the stimuli. In other worlds, it is not clear whether the stimulus duration contrast indicates the neural correlates of how participants *perceived* time differently, or whether the neural effect was driven by the longer presentation times. There is evidence consistent with the latter explanation, since in the contrast of increasing stimulus duration in Study 1 we identified activation in the visual cortex, which could be explained by a purely sensory account.

This confound was therefore eliminated in Study 2 where participants had to judge the duration of a fixed duration stimulus. This was a stimulus duration tailored to each participant’s BP where they responded equally frequently to “short” or “long” (calculated from a calibration task similar to Study 1) and thus any neural differences found in this contrast corresponds to how participants *perceived* time, free of any confounds of the *actual* duration of the stimuli (which was identical throughout). In Study 2 we did not identify any visual cortex activation, consistent with the above explanation.

We also failed to detect any correlations between (i) the behavioural effect of threat and the neural effect of threat>safe and (ii) the behavioural effect of threat and neural activation in the mid-cingulate area that was active during both the threat>safe and the perceived long>perceived short contrasts (exploratory analyses). This may be due to low statistical power, as our study was not optimized to examine individual differences. Indeed, in order to detect a correlation of .30 with 0.80 power we would need 82 participants, which was beyond the scope of the current within-subjects design.

It should also be noted that our fMRI paradigm does not allow us to dissociate between perception of the stimulus and the response, thus all interpretations should be made with this in mind. A future study could further delineate this by introducing a jittered delay between stimuli perception and task response in the design of the task. This (longer) design would enable the investigation of how anxiety affects time perception at the stage of perception and/or decision and which neural circuits are involved at each stage. On a related note, we attempted to account for the delivery of shocks in the fMRI modelling, and in some threat blocks there were no shocks, but it is difficult to fully disentangle the effect of shock anticipation from the direct effect of shocks themselves.

Finally, we tested healthy individuals under an anxiety manipulation. Whether our results would generalize to pathological anxiety remains an open question.

Future work directly comparing induced anxiety with non-affective load manipulations within the same paradigm, and using designs that separate perceptual from decisional stages, will be crucial for adjudicating between resource-overloading, affective modulation, and sensory-loss accounts of anxiety-induced temporal distortion.

## Conclusion

We replicated previous findings of temporal underestimation in anxiety, and found activation in brain areas previously associated with threat-of-shock-induced anxiety (sgACC, insula, caudate) and time perception (mid-cingulate cortex). Despite previous studies suggesting a key role of pre-SMA in temporal perception, the mid-cingulate cortex was more robustly activated in our study. We suggest a potential role of the mid-cingulate cortex in mediating emotion-related alterations in temporal perception. Interestingly, we found evidence of overlap between activations elicited by time perception and anxiety (insula and mid-cingulate cortex), which is consistent with the hypothesis that anxiety may influence cognition by further loading already-in-use resources.

## Supplementary Material

nsag006_Supplementary_Data

## Data Availability

The behavioural data generated during the current study are available in the figshare repository https://figshare.com/s/26e4d5a4fd2d23421b13. The functional imaging datasets generated during the current study are not publicly available because they cannot be safely anonymized. They can be accessed from the senior author O. Robinson (o.robinson@ucl.ac.uk) upon reasonable request with a data-sharing agreement in place.
