# Supplementary material for: Anxiety makes time pass quicker: neural correlates
Source: Soc Cogn Affect Neurosci. 2026 Feb 6;21(1):nsag006. doi: 10.1093/scan/nsag006 (PMC13089397; doi:10.1093/scan/nsag006)
Supplement: nsag006_Supplementary_Data [file nsag006_supplementary_data.zip › Table S2.docx]

Table S2: fMRI activation overlap between Study 1 & 2 for threat>safe.

|  |  | MNI coordinates | | |  |  | cluster | peak |
| --- | --- | --- | --- | --- | --- | --- | --- | --- |
| region | hemisphere | x | y | z | #voxels | Zvalue | p(FWE-corr) | p(FWE-corr) |
| insula | right | 33 | -1 | -10 | 11 | 4.51 | 0.754 | 0.003 |
| white matter | right | 18 | 38 | 5 | 1 | 4.25 | 0.911 | 0.009 |
| lateral caudate nucleus | right | 18 | 20 | 17 | 49 | 4.10 | 0.354 | 0.017 |
| white matter | right | 27 | 20 | 23 | “ | 4.04 | “ | 0.022 |
