# Supplementary material for: Anxiety makes time pass quicker: neural correlates
Source: Soc Cogn Affect Neurosci. 2026 Feb 6;21(1):nsag006. doi: 10.1093/scan/nsag006 (PMC13089397; doi:10.1093/scan/nsag006)
Supplement: nsag006_Supplementary_Data [file nsag006_supplementary_data.zip › Supplementary material v2 submitted.docx]

# Supplementary material

# Experiment specific methods

## Study 1: Temporal bisection task under threat of shock

The fMRI study and all procedures were approved by the NIH Institutional Review Board Project (ID Number: 02-M-0321) and were conducted in accordance with the Declaration of Helsinki. The aim of Study 1 was to identify regions of interest (ROIs); 13 individuals were scanned.

Participants were recruited through advertisements (newspaper and public transport) in the Washington, D.C. metropolitan area. Following an initial telephone screen, individuals visited the NIH for comprehensive screening by a clinician, which comprised a physical examination, urine drug screen, screen for MRI suitability, and the Structured Clinical Interview for the Diagnostic and Statistical Manual of Mental Disorders, Fifth Edition (American Psychiatric Association et al., 2013). Exclusion criteria were: contraindicated medical disorder (i.e. those thought to interfere with brain function and/or behaviour); past or current psychiatric disorders; and use of psychoactive medications or recreational drugs (per urine screen). All participants provided written informed consent and were reimbursed for their participation. As this was an exploratory pilot experiment to inform Study 2, no *a priori* power calculation was performed.

In Study 1, the duration of the to-be-timed stimuli varied between 1.4-2.6s (six possible durations: 1.4s, 1.64s, 1.88s, 2.12s, 2.36s, and 2.6s). The session consisted of two runs, each comprising of four blocks, two in the safe and two in the threat condition (counterbalanced) with 36 trials per block.

Seventy-two pictures were used in this experiment, depicting happy, neutral and fearful facial expressions, taken from 24 actors. During each block, participants viewed an equal number of happy, fearful and neutral facial expressions, the order of which was pseudorandomised. Similarly, stimulus durations were pseudorandomised within each block, so that all durations were repeated six times.

Participants received between 0-3 shocks during each threat block, only during threat blocks. The order of the shocks was random for each participant and occurred on different trials, following the participants’ response.

Scanning was performed on a 3T Siemens Magnetom Skyra using a 32-channel head coil. Each echo planar imaging (EPI) volume consisted of 34 slices and was acquired in 2s (repetition time: TR) using a 2D sequence. Each scan was preceded by dummy scans which were discarded. Other parameters of the functional EPI were the following: voxel size of 3mm*3mm (slice thickness of 2.5mm, 0.5mm slice separation), field of view=216mm*216mm, echo time (TE)=30ms; flip angle=70°.

## Study 2: Modified temporal bisection task under threat of shock

The studies and all procedures were approved by the UCL Research Ethics Committee (Project ID Number: 1227/001) and were conducted in accordance with the Declaration of Helsinki. Prior to the fMRI study we completed a behavioural pilot to ensure that the properties of the modified task were as expected. We pre-registered this study https://osf.io/54qfh (Sarigiannidis, 2019).

Participants were recruited from UCL databases. A telephone interview was conducted to screen for past neurological or psychiatric diagnosis and to determine MRI safety. In addition, on the day of testing participants completed self-report measures of depression (Beck Depression Inventory: BDI; (Beck and Steer, 1987)) and trait anxiety (State Trait Anxiety Inventory: STAI; (Spielberger, 1983) to ensure they fell within the non-clinical range. Participants provided written informed consent and received £20 for the fMRI study (which lasted approximately two hours: one hour for task explanation and questionnaires, and one hour for scanning).

A power calculation (G*power version 3.1.9.2 (Faul et al., 2007)) determined the sample size based on the meta-analytic effect of threat (d=0.68) from our previous behavioural temporal bisection studies (Sarigiannidis et al., 2020). Given that the MRI scanner may be an anxiogenic environment, which could raise anxiety levels during the safe condition and therefore reduce condition differences, we decreased the expected effect size by ~15% (d=0.47). To achieve d=0.47 with 80% power at alpha=0.05 (one-tailed due to directional prediction), the required sample size required was 30 participants. In the fMRI study, one participant was excluded from analysis as they pressed the same button on every trial, leaving a final sample size of 29.

Before the main task, participants performed the calibration phase in which the trial structure was identical to that of Study 1, but consisted only of a single safe block of 72 trials. This data was analysed immediately after completion of the calibration task in order to calculate the BP for each participant (i.e. the duration for which participants responded “short” or “long” equally often). This was set as the main stimulus duration for the fMRI experiment, in order to remove a potential confound in Study 1: in that design, it was not clear whether the stimuli duration contrast reflected the neural correlates of how participants *perceived* time differently, or whether the neural effect was driven by the *actual duration differences* between the stimuli. In order to minimise scanning time, participants performed this calibration task while anatomical brain images were acquired.

During the main task, each of the two runs consisted of six blocks (three safe and three threat blocks per run). Each block comprised 18 trials: the stimulus duration of 14 trials was set to the BP of each participant (calculated from the calibration task), the stimulus duration of two trials was 1.4s (the short “anchor” duration) and the stimulus duration of two trials was 2.6s (the long “anchor” duration). Thus, although in Study 1 and in the calibration task participants viewed six different durations (1.4s, 1.64s, 1.88s, 2.12s, 2.36s, 2.6s), in the main task for Study 2 they viewed just three (1.4s, BP, 2.6s). Participants were instructed that in the main task the stimuli durations would be more difficult to tell apart compared to the calibration task. At the end of each block of the main task, a 20s rest period followed during which the screen went blank and participants were instructed to rest while the scan finished.

Participants received between zero and three shocks per block during the threat condition, according to a pre-determined schedule. The order of the shocks was random for each participant and occurred on different trials during the ITI. Each train of shocks consisted of 10 pulses delivered over 0.5s.

Scanning was performed on a 3T Siemens Magnetom Prisma using a 64-channel head coil. The 1mm isotropic anatomical scan was a T1-weighted MPRAGE with the following parameters: TR=2.53s, TE=3.34ms, acquisition matrix=256 x 256, slice thickness=1mm, flip angle=7^o^. EPI scans were acquired with a 2D sequence. Each volume consisted of 42 slices and was acquired in TR=2.94s. The angulation of the slice (T>C-30°), the phase-encoding direction and the compensating gradients (z-shimming) were optimized to minimize the dropout in regions near the orbito-frontal cortex and amygdalae (Weiskopf et al., 2006). Each scan was preceded by dummy scans which were discarded. Other parameters of the EPI were: voxel size 3mm*3mm in plane (slice thickness 2.5mm, 0.5mm slice separation); field of view 192mm*192mm; 12% over-sampling in the phase-encoded direction; bandwidth=2298 Hz/px; echo spacing=0.5ms, TE=30ms; flip angle=90°. Fat saturation with an excitation of 130° was used prior to each excitation. At the end of each session, we acquired one fieldmap with identical parameters to the EPI scans. Heart rate and breathing were monitored using Spike2 software (<http://ced.co.uk/products/spkovin>).

For the Study 2 we have reported all measures, conditions, data exclusions, and how we determined our sample sizes. It should be noted that the pre-registration erroneously states that frontopolar activations were identified in Study 1 (Figure S1), which was not the case.

## Behavioural data analysis

All data was processed in Matlab (v. R2015b), and statistical analysis was carried out in SPSS (v. 23).

### Study 1

Trials on which participants did not make a response were excluded from the analysis. Repeated-measures analyses of variance (ANOVAs) were performed on the proportion of stimuli participants judged to be long (p(long)). Threat (safe or threat-of-shock condition) and duration (six stimulus durations) were within-subject factors. Greenhouse-Geisser corrections were applied when violations of sphericity occurred. The BP was calculated for safe and threat conditions separately for each participant and the effect of threat was assessed using a paired-samples t-test.

### Study 2

Trials on which participants did not make a response were excluded from the analysis. As the participants were only shown one intermediate stimulus at their BP, instead of the ANOVA used in the previous design, a paired-samples t-test was used to test the effect of threat on the proportion of long responses made at each participant’s BP (N=84 trials per condition). We did not analyse catch trials, i.e. the few trials (N=4 per block) whose durations were equal to the “short” (1.4s) and “long” (2.6s) anchors. However, we did use them to exclude one participant who pressed the same button on all trials, including both anchors, indicating that they did not comply with the instructions.

## Functional neuroimaging data analysis

### Study 1

Trials were modelled as events of zero duration. The regressors of interest were the onsets of the to-be-timed stimuli of the six durations (1.4s, 1.64s, 1.88s, 2.12s, 2.36s, 2.6s) during safe and threat blocks (i.e. 12 regressors of interest). Regressors of no interest were the training stimuli indicating the anchor durations (presented before the beginning of each block), shocks, as well as the start screens of each block (indicating whether in that block participants were safe or under threat of shock). All these regressors were convolved with SPM's canonical hemodynamic response function, time-locked to the onset of the corresponding event. We also included six movement regressors of no interest in all participants.

Using the general linear model, parameter estimate images were created for each regressor, and combined to create the primary contrasts at the subject level.

Second-level analyses were conducted using the standard summary statistics approach to random effects analysis. In this exploratory pilot study, we applied a lenient cluster-forming threshold of p<0.05 (uncorrected) cluster size>150, but did not use this to make inference. Instead we used the resulting clusters as ROIs in Study 2.

The fMRI contrasts were: 1) the effect of the threat compared to the safe condition; 2) the effect of stimulus duration, which was a linear contrast across the six stimulus durations (from short to long), collapsed across the safe and threat conditions; 3) the interaction between duration and threat.

# Results

## Study 1

### Behavioural results inside the scanner

Across blocks, participants reported being significantly more anxious in the threat compared to the safe condition (t(12)=5.40, p<0.001, d=1.56). There was a significant effect of stimulus duration (F(2.46, 29.58)=211.51, p<0.001, η_p_^2^=.946; see Figure 2). As expected, the longer the stimulus duration, the more likely it was to be classified as “long”. The effect of threat was non-significant (F(1, 12)=2.57, p=0.135, η_p_^2^=.177) as was the threat-by-duration interaction (F(5, 60)=0.27, p=0.926, η_p_^2^=.022). The BP was not significantly different between the threat and safe conditions (t(12)=1.36, p=0.194, d=0.37), although the direction of the effect was consistent with our previous studies (a rightward shift in the psychometric function, consistent with temporal underestimation during threat). This non-significant result is unsurprising since the experiment was underpowered to detect the magnitude of the behavioural effect we identified previously (d=68), and we emphasise that testing this behavioural hypothesis was not the purpose of Study 1.

### Neural effect of threat

No voxels survived correction for multiple comparisons in the threat contrast, the duration contrast or the interaction. Therefore, the below analyses were conducted using an exploratory threshold of P<0.05 (uncorrected), and used to generate hypotheses for Study 2. Only large clusters (k>150) are reported (see Table S1)

#### Threat>safe

This analysis examined the effect of the threat-of-shock vs the safe condition and revealed activations in two large clusters. One cluster was in the parietal cortex, consisting mainly of white matter and therefore was not considered further. The peak activation in the other cluster was in the left caudate ([x=-18, y=11, z=26) while in the same cluster, there was also a sub-peak in mid-cingulate cortex ([x=0, y=-4, z=50]); see Figure S1A. Both of these peaks were used as the centres of ROIs for the threat contrast in Study 2.

#### Safe>threat

This analysis examined the effect of the safe vs the threat-of-shock condition and revealed activations in one large cluster, with a peak in the right supramarginal gyrus.

### Neural effect of stimulus duration

#### Long>short

This analysis examined the effect of stimulus duration implementing a linear contrast (-2.5, -1.5, -0.5, 0.5, 1.5, 2,5 corresponding to the six stimulus durations: 1.4s, 1.64s, 1.88s, 2.12s, 2.36s, 2.6s) and revealed activation in one large cluster, with a peak activation in the visual cortex (right superior occipital gyrus; see Figure S1B).

#### Short>long

This analysis examined the effect of stimulus duration implementing the inverse of the above contrast which revealed activation in one large cluster, with a peak activation in the left superior cerebellar peduncle.

### Neural effect of threat × stimulus duration interaction

The analysis examined the interaction of the above effects, revealing activation in one large cluster, with a peak activation in the precuneus. There was also activation in the frontal areas, specifically in the left middle frontal gyrus (see Figure S1C). The inverse contrast revealed activation in one large cluster, with a peak activation in the precentral gyrus.

**Overlap between Study 1 & 2**

A whole-brain mask (thresholded at p<0.05 uncorrected) was created from each contrast of Study 1 (using the ImCalc function in SPM) and was applied to the corresponding contrast for Study 2, using P<0.005 as the cluster-forming threshold and a 10-voxel cluster size. Statistical inference was based on the peak level statistics, with voxel-level small volume correction applied across the mask.

When applying a mask generated from the threat > safe contrast in Study 1 to the equivalent contrast in Study 2 there was significant (voxel-level FWE corrected) activation in the right insula and the right caudate. There was no significant overlap between Studies 1 & 2 for the duration or interaction contrasts.

**ROI extraction for threat-by-perceived duration ANOVA in study 2**

To test our hypothesis that anxiety may “overload” regions processing time perception, we performed a 2-by-2 (threat-by-perceived duration) ANOVA on average activation across the ROIs in Figure S3. However, there was no significant interaction between threat and perceived duration for either the insula (F(1,28)=.049, p=.827) or the mid-cingulate (F(1,28)=.075, p =.786). We report only these interactions here, and not the main effects, to avoid circularity in the analysis. The main effects of threat and perceived durations have already been reported in previous sections.

One possibility is that individuals whose task responses were affected by anxiety (i.e. underestimated time), would show an interaction between the insula and the mid-cingulate, due to neural overloading. At the same time, we would not expect an interaction on individuals who did not underestimate time under threat. Hence, we extracted the activation of the insula and mid-cingulate areas from the interaction contrast, and correlated it with the degree of temporal underestimation during threat. No significant correlations were observed, either between the effect of threat on pLong and the right insula from the interaction contrast (r(29)=.16, p=.51), or between the effect of threat on pLong and the mid-cingulate from the interaction contrast (r(29)=.07, p=.72).

Correlation of threat-of-shock-based activation (threat *minus* safe) with behavioural threat-of-shock effect (pShort for threat condition *minus* pShort for safe condition)

|  | Threat minus safe contrast | Interaction contrast |
| --- | --- | --- |
| ACC | r = -0.273, p = 0.152 | r = 0.069, p = 0.723 |
| Insula | r = -0.130, p = 0.503 | r = 0.125, p = 0.517 |

# References

American Psychiatric Association, American Psychiatric Association, DSM-5 Task Force, 2013. Diagnostic and statistical manual of mental disorders: DSM-5. American Psychiatric Association, Washington, D.C.

Beck, A.T., Steer, R.A., 1987. BDI, Beck depression inventory: manual. Psychological Corp. ; Harcourt Brace Jovanovich, San Antonio, Tex.; New York.

Faul, F., Erdfelder, E., Lang, A.-G., Buchner, A., 2007. G*Power 3: A flexible statistical power analysis program for the social, behavioral, and biomedical sciences. Behav. Res. Methods 39, 175–191. https://doi.org/10.3758/BF03193146

Sarigiannidis, I., 2019. The functional neural correlates of time perception under threat of shock. https://doi.org/None

Sarigiannidis, I., Grillon, C., Ernst, M., Roiser, J.P., Robinson, O.J., 2020. Anxiety makes time pass quicker while fear has no effect. Cognition 197, 104116. https://doi.org/10.1016/j.cognition.2019.104116

Spielberger, C.D., 1983. Manual for the State-Trait Anxiety Inventory STAI (Form Y) (“Self-Evaluation Questionnaire”).

Weiskopf, N., Hutton, C., Josephs, O., Deichmann, R., 2006. Optimal EPI parameters for reduction of susceptibility-induced BOLD sensitivity losses: a whole-brain analysis at 3 T and 1.5 T. NeuroImage 33, 493–504. https://doi.org/10.1016/j.neuroimage.2006.07.029

**Figure legends:**

**Figure S1**: *Uncorrected, exploratory BOLD activation for each contrast in Study 1: Left) threat>safe; Center) linear effect of lengthening temporal interval; Right) positive interaction of A and B. A threshold of P<0.05 (uncorrected) was used, colour bars indicate t-values.*

**Figure S2**: *BOLD co-activations for threat>safe contrasts for Study 1 and Study 2. Figure generated by creating masks from the threat>short contrast for Studies 1 & 2, thresholded at t>1.78 and t>2.76 respectively. Left panel: insula, right panel: caudate*

**Figure S3**: *Averaged activation across the ROI for each condition in the right insula (top panel) and the mid-cingulate (bottom panel). Errors bars are standard errors.*
