# Supplementary material for: Anxiety makes time pass quicker: neural correlates
Source: Soc Cogn Affect Neurosci. 2026 Feb 6;21(1):nsag006. doi: 10.1093/scan/nsag006 (PMC13089397; doi:10.1093/scan/nsag006)
Supplement: nsag006_Supplementary_Data [file nsag006_supplementary_data.zip › Table S1.docx]

Table S1: Activations for the Study 1 contrasts, exploratory cluster forming threshold p<0.05 (uncorrected)

|  |  |  | MNI coordinates | | |  |  | cluster | peak |
| --- | --- | --- | --- | --- | --- | --- | --- | --- | --- |
| contrast | region | hemisphere | x | y | z | #voxels | Zvalue | p(FWE-corr) | p(FWE-corr) |
| threat>safe | white matter | n/a | 0 | 27 | -7 | 390 | 3.51 | 0.136 | 1 |
|  | caudate | left | -18 | 11 | 26 | 240 | 3.26 | 0.626 | 1 |
|  | anterior cingulate | n/a | 0 | -4 | 50 | “ | 2.51 | “ | 1 |
| safe>threat | supramarginal gyrus | right | 36 | -43 | 35 | 272 | 3.77 | 0.471 | 0.997 |
| long>short | occipital gyri | right | 24 | -100 | 8 | 247 | 3.47 | 0.989 | 0.992 |
| short>long | superior cerebellar peduncle | left | -3 | -25 | -13 | 24626 | 4.56 | <0.001 | 0.154 |
| interaction | precuneus | left | -9 | -67 | 44 | 446 | 2.63 | 0.491 | 1 |
|  | frontomarginal gyrus | left | -24 | 46 | 2 | 66 | 3.15 | 1 | 1 |
| interaction (inverse) | precentral gyrus | right | 59 | -2 | -23 | 182 | 2.88 | 0.997 | 1 |
