# Supplementary figures and images for: Anxiety makes time pass quicker: neural correlates
Source: Soc Cogn Affect Neurosci. 2026 Feb 6;21(1):nsag006. doi: 10.1093/scan/nsag006 (PMC13089397; doi:10.1093/scan/nsag006)

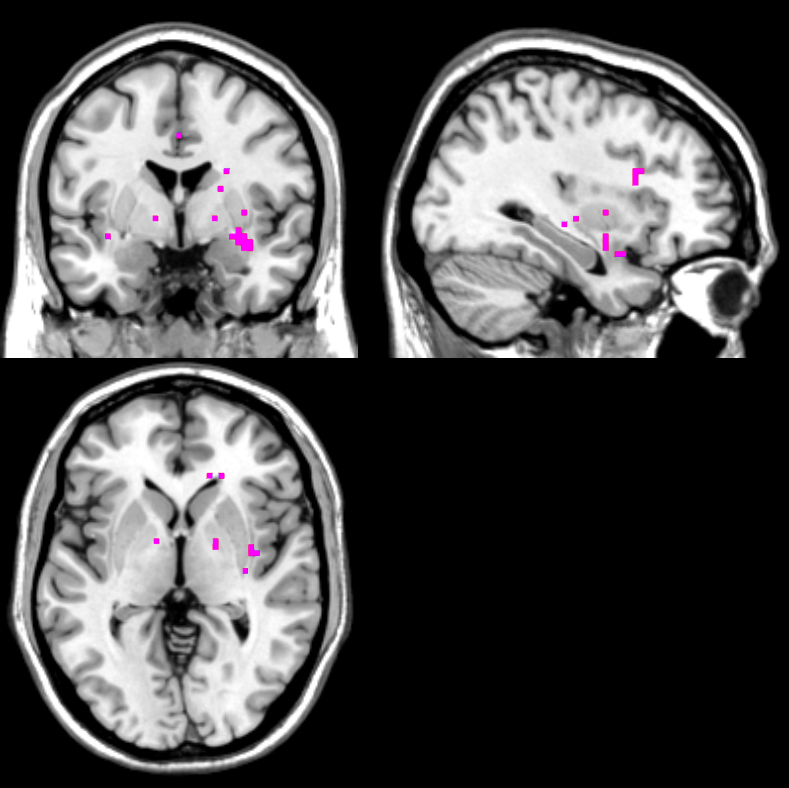

Supplement: nsag006_Supplementary_Data [file nsag006_supplementary_data.zip › SFigure 2.jpg]

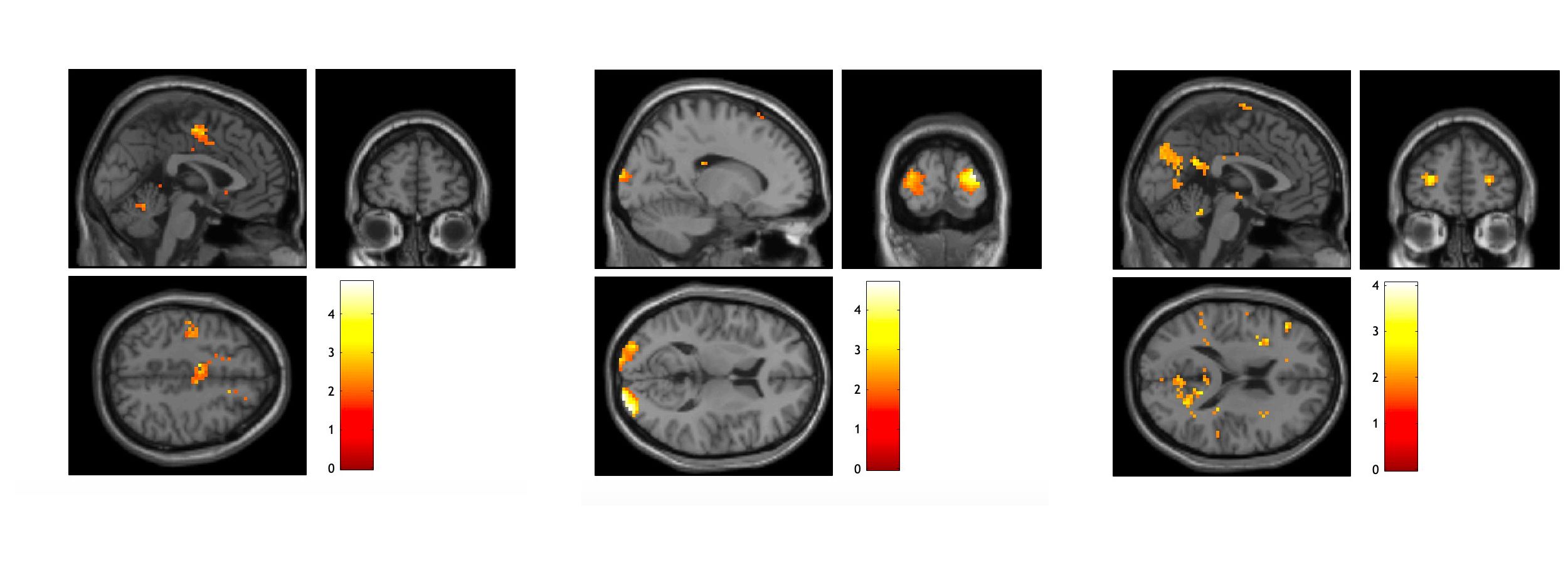

Supplement: nsag006_Supplementary_Data [file nsag006_supplementary_data.zip › SFigure1.jpg]
